# Antifungal Activity of Isavuconazole and Comparator Agents against Contemporaneous Mucorales Isolates from USA, Europe, and Asia-Pacific

**DOI:** 10.3390/jof9020241

**Published:** 2023-02-11

**Authors:** Cecilia G. Carvalhaes, Paul R. Rhomberg, Michael D. Huband, Michael A. Pfaller, Mariana Castanheira

**Affiliations:** JMI Laboratories, 345 Beaver Kreek Centre, North Liberty, IA 52317, USA

**Keywords:** *Rhizopus*, *Rhizomucor*, *Lichtheimia*, azoles, antifungal activity, mucormycosis

## Abstract

Isavuconazole is the only US FDA-approved antifungal for treating invasive mucormycosis. We evaluated isavuconazole activity against a global collection of Mucorales isolates. Fifty-two isolates were collected during 2017–2020 from hospitals located in the USA, Europe, and the Asia-Pacific. Isolates were identified by MALDI-TOF MS and/or DNA sequencing and susceptibility tested by the broth microdilution method following CLSI guidelines. Isavuconazole (MIC_50/90_, 2/>8 mg/L) inhibited 59.6% and 71.2% of all Mucorales isolates at ≤2 mg/L and ≤4 mg/L, respectively. Among comparators, amphotericin B (MIC_50/90_, 0.5/1 mg/L) displayed the highest activity, followed by posaconazole (MIC_50/90_, 0.5/8 mg/L). Voriconazole (MIC_50/90_, >8/>8 mg/L) and the echinocandins (MIC_50/90_, >4/>4 mg/L) had limited activity against Mucorales isolates. Isavuconazole activity varied by species and this agent inhibited at ≤4 mg/L 85.2%, 72.7%, and 25% of *Rhizopus* spp. (*n* = 27; MIC_50/90_, 1/>8 mg/L), *Lichtheimia* spp. (*n* = 11; MIC_50/90_, 4/8 mg/L), and *Mucor* spp. (*n* = 8; MIC_50_, >8 mg/L) isolates, respectively. Posaconazole MIC_50/90_ values against *Rhizopus*, *Lichtheimia*, and *Mucor* species were 0.5/8 mg/L, 0.5/1 mg/L, and 2/- mg/L, respectively; amphotericin B MIC_50/90_ values were 1/1 mg/L, 0.5/1 mg/L, and 0.5/- mg/L, respectively. As susceptibility profiles varied among Mucorales genera, species identification and antifungal susceptibility testing are advised whenever possible to manage and monitor mucormycosis.

## 1. Introduction

The epidemiology of mucormycosis has changed over the last few decades. Although it is still considered a rare disease, the incidence of mucormycosis is increasing worldwide, especially in developing countries [1]. This increase is primarily due to the expansion of patient populations most at risk for mucormycosis and the increased use of prophylactic antifungal agents to prevent invasive fungal infections (IFIs) [1,2]. Patients undergoing hematopoietic stem cell or solid organ transplantation, as well as patients with uncontrolled diabetes mellitus, are at particular risk [3,4]. Although prophylactic antifungal agents are recommended for patients undergoing hematopoietic stem cell or solid organ transplantation to prevent IFIs, breakthrough mucormycosis cases still occur due to limitations in the activity of most antifungal agents against Mucorales organisms [5,6]. Furthermore, in the past two years, clinicians have witnessed a dramatic increase in reports of mucormycosis associated with SARS-CoV-2, corticosteroid use, and uncontrolled diabetes mellitus in some regions [7,8,9].

Liposomal amphotericin B is the first-line treatment in the management of mucormycosis and is recommended by the European Confederation of Medical Mycology and the International Society for Human and Animal Mycology (ECMM/ISHAM), as well as other scientific associations. However, posaconazole and isavuconazole are usually used as salvage treatment in cases of mucormycosis that have poor response to amphotericin B [1,9,10].

Isavuconazole is a novel extended spectrum triazole with activity against yeasts, moulds, including Mucorales, and dimorphic fungi [11]. Similar to other azoles, isavuconazole inhibits cytochrome P450 (CYP)-dependent 14 α-lanosterol demethylation, which is essential for fungal membrane ergosterol synthesis. This inhibition ultimately leads to the accumulation of toxic sterols and cell death. Isavuconazonium sulfate, the water-soluble prodrug, is rapidly hydrolyzed to the triazole isavuconazole after intravenous or oral administration, with high oral bioavailability [12]. Isavuconazole is the only US Food and Drug Administration (FDA)-approved antifungal agent for treating invasive mucormycosis [13]. The purpose of the present survey is to provide in vitro data on the activity of isavuconazole and comparator agents against contemporary Mucorales isolates causing invasive infections worldwide, using the reference Clinical Laboratory Standard Institute (CLSI) broth microdilution method. Isolates were collected from three global regions during 2017–2020.

## 2. Materials and Methods

### 2.1. Fungal Isolates

A total of 52 non-duplicate Mucorales isolates causing invasive infections were collected from 20 medical centers located in North America (21 isolates from 11 centers in USA), Europe (23 isolates from 6 centers in 5 countries: France, Germany, Slovenia, Sweden, and Turkey), and the Asia-Pacific region (8 isolates from 3 medical centers in 3 countries: Australia, Thailand, and South Korea). Participating medical centers submitted consecutively collected fungal isolates, deemed by local criteria to cause invasive infections, to a central monitoring laboratory (JMI Laboratories, North Liberty, Iowa, USA) as part of the 2017–2020 SENTRY Antifungal Surveillance Program. Only a single isolate per patient was included. Fungal isolates were collected from pneumonia in hospitalized patients (28 isolates; 53.8%), skin and skin structure infections (15 isolates; 28.8%), and other non-specified sites (9 isolates; 17.3%).

Fungal isolates were identified by matrix-assisted laser desorption ionization-time of flight mass spectrometry (MALDI-TOF MS; Bruker Daltonics, Bremen, Germany) using MALDI MBT Compass 4.1.100, Filamentous Fungi Library 3.0, and the proprietary library of JMI Laboratories. The JMI proprietary library includes 63 filamentous fungi isolates, including 29 species and species complex, and 11 isolates only identified at genus level. Only 4 isolates within the Mucorales order, comprising 2 genera (*Rhizopus* and *Cunninghamella*), were included in the JMI proprietary library. Isolates that did not score ≥2.0 by spectrometry were submitted to confirmatory identification by sequencing and analysis of the ITS and/or 28S ribosomal subunit [14,15]. Nucleotide sequences were analysed using Lasergene^®^ software (DNAStar, Madison, WI, USA) and compared to available sequences with BLAST (https://blast.ncbi.nlm.nih.gov/Blast.cgi (accessed on 7 February 2023)). Results were considered acceptable if the homology was ≥99.5% with other entries in the databases used for comparison.

### 2.2. Antifungal Susceptibility Testing

Isolates were susceptibility tested by the broth microdilution method following the guidelines in the CLSI M38 [16,17] document. The following antifungal agents were included in this study: isavuconazole, itraconazole, posaconazole, voriconazole, amphotericin B, anidulafungin, caspofungin, and micafungin. Quality control was performed and interpreted as recommended by the CLSI M38M51S (2022) document using *C. krusei* ATCC 6258, *C. parapsilosis* ATCC 22019, *Aspergillus flavus* ATCC 204304, *A. fumigatus* MYA-3626, and *Hamigera insecticola* ATCC MYA-3630 [18]. No CLSI clinical breakpoints or epidemiological cut-off values were available for these organisms.

## 3. Results

**Figure 1:**
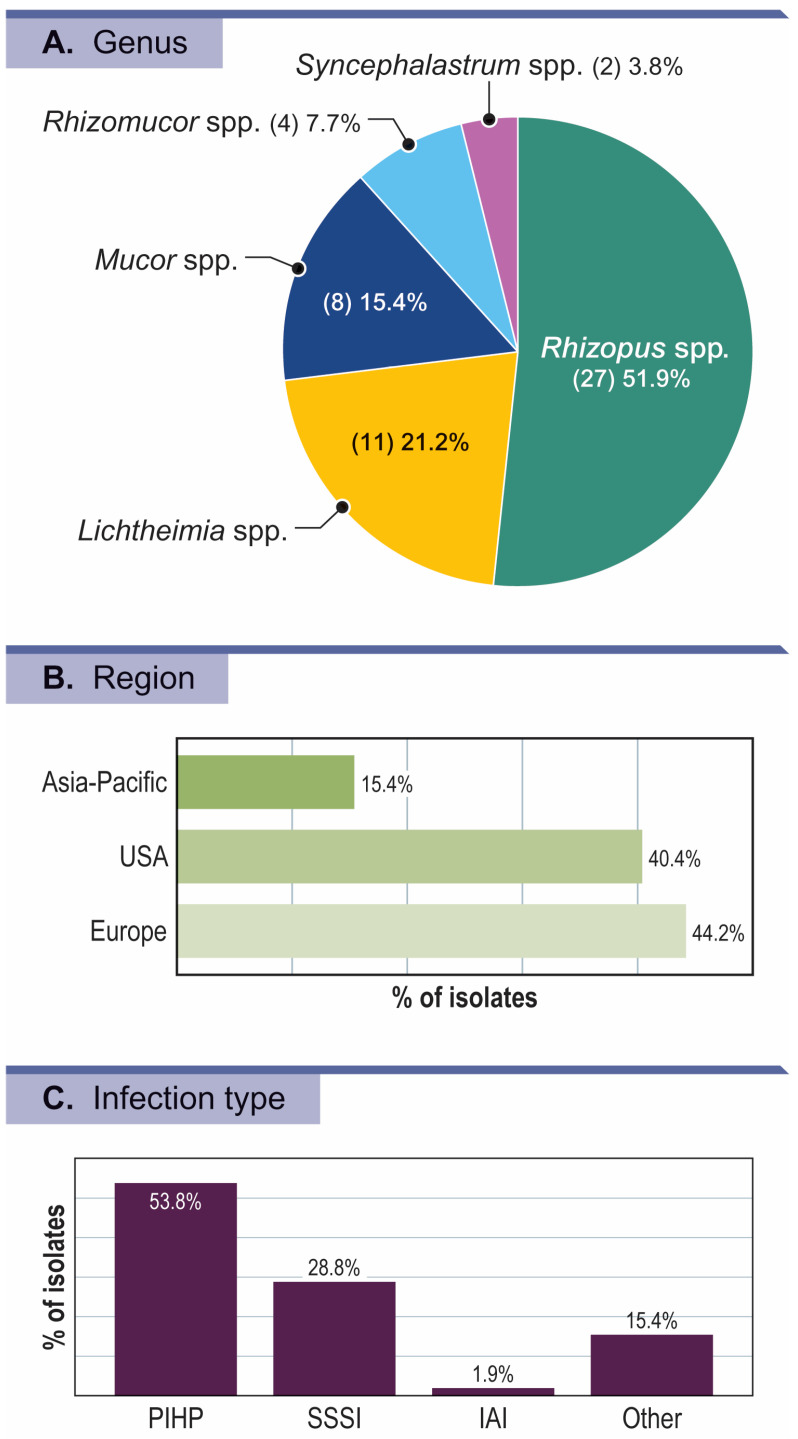
Distribution of Mucorales isolates recovered from invasive fungal infections worldwide (2017–2020). PIHP, pneumonia in hospitalized patients; SSSI, skin and skin structure infection; IAI, intra-abdominal infection.

The Mucorales clinical isolates collected and tested in surveillance years 2017–2020 are presented in Figure 1A. Overall, *Rhizopus* (27 isolates; 51.9% of all Mucorales) was the most frequently isolated genera, followed by *Lichtheimia* (11 isolates; 21.2%), *Mucor* (eight isolates; 15.4%), *Rhizomucor* (four isolates; 7.7%), and *Syncephalastrum* (two isolates; 3.8%; Table 1). Of the 52 clinical isolates tested, 37 (71.2%) were identified by MALDI-TOF MS: 33 (63.5%) to the species level and two to the genera level (one *Rhizopus* spp. and one *Lichtheimia* spp.). For two *Mucor* isolates, MALDI-TOF MS could not differentiate between *M. circinelloides* and *M. ramosissimus*. The 15 remaining isolates were identified by ITS and/or 28S rDNA sequencing; nine of which were identified to the species or species complex, including two *Lichtheimia corymbifera*, one *Mucor indicus*, one *Rhizomucor pusillus*, one *Rhizopus microsporus* group, two *Rhizopus arrhizus*, and two *Rhizopus arrhizus* species complex. Six isolates were not resolved to the species or species complex level using ITS sequencing—two *Lichtheimia* spp., two *Mucor* spp., and two *Syncephalastrum* spp. Isolates were mainly from Europe (44.2%) and USA (40.4%), with a few isolates recovered from Asia-Pacific (*n* = 8, 15.4%; Figure 1B). The genera distribution was similar among regions, except that *Lichtheimia* spp. was more frequently recovered from Europe (seven Europe, three USA, one Asia-Pacific), and mainly from Slovenia (six/seven isolates; Table S1).

### Activity of Isavuconazole and Comparators against Mucorales Isolates

The cumulative distribution MIC values for isavuconazole and comparator agents against Mucorales isolates, including data for *Lichtheimia* spp., *Mucor* spp., *Rhizomucor* spp., *Rhizopus* spp., and *Syncephalastrum* spp., is displayed in Table 1. The activity of isavuconazole and comparator agents against the Mucorales order was split by genera and species and are displayed in Table 2.

Isavuconazole (MIC_50/90_, 2/>8 mg/L) inhibited 59.6% and 71.2% of all Mucorales isolates at ≤2 mg/L and ≤4 mg/L, respectively. This agent retained similar activity against isolates from the USA (21 isolates; MIC_50/90_, 2/>8 mg/L), Europe (23 isolates; MIC_50/90_, 2/8 mg/L), and Asia-Pacific (eight isolates; MIC_50_, 2 mg/L), with a total of 76.2%, 73.9% and 50.0% of Mucorales isolates inhibited by isavuconazole at ≤4 mg/L, respectively. Overall, the most active antifungal agents against the Mucorales order were amphotericin B (MIC_50/90_, 0.5/1 mg/L), followed by posaconazole (MIC_50/90_, 0.5/8 mg/L), isavuconazole (MIC_50/90_, 2/8 mg/L), and itraconazole (MIC_50/90_, 2/8 mg/L; Table 2). Limited activity was displayed by voriconazole (MIC_50/90_, >8/>8 mg/L; Table 2) and the echinocandins (MEC_50/90_, >4/>4 mg/L; data not shown).

*Rhizopus* spp. was the most frequent genera recovered (51.9%; 27/52 isolates), including 16 *R. microsporus* group, 10 *R. arrhizus* species complex, and one *Rhizopus* spp. Isavuconazole was active against all *Rhizopus* spp. (MIC_50/90_, 1/>8 mg/L) and particularly active against *R. microsporus* group (MIC_50/90_, 1/2 mg/L), inhibiting all 16 isolates at ≤4 mg/L. Isavuconazole (MIC_50/90_, 1/>8 mg/L) exhibited similar activity to posaconazole (MIC_50/90_, 0.5/>8 mg/L) and itraconazole (MIC_50/90_, 2/>8 mg/L) against all *Rhizopus* spp. isolates. However, isavuconazole displayed lower MIC_90_ values (MIC_90_, 2 mg/L) than posaconazole (MIC_90_, >8 mg/L) and itraconazole (MIC_90_, >8 mg/L) against *R. microsporus* group. All *Rhizopus* spp. isolates were inhibited by amphotericin B at ≤2 mg/L.

*Lichtheimia* spp. (11 isolates, 21.2%) was the second most common genera, with 7 of 11 isolates recovered from Europe (all from Slovenia), three from USA, and only one from Asia-Pacific (Australia). The majority of *Lichtheimia* isolates were confirmed as *L. corymbifera* (7/11; 63.6%). Two of three *Lichtheimia* spp. could not be differentiated between *L. corymbifera* and *L. ramosa* by ITS and 28S rDNA sequencing. Isavuconazole (MIC_50/90_, 4/8 mg/L) inhibited 8/11 (72.7%) *Lichtheimia* spp. isolates, including six *L. corymbifera* and two *Lichtheimia* spp., at ≤4 mg/L. Amphotericin B (MIC_50/90_, 0.5/1 mg/L), posaconazole (MIC_50/90_, 0.5/1 mg/L), and itraconazole (MIC_50/90_, 1/2 mg/L) showed equivalent activity against *Lichtheimia* spp. isolates. Voriconazole showed poor activity against these isolates (MIC_50/90_, >8/>8 mg/L).

*Mucor* spp. was the third most frequent genera recovered, with only eight representative isolates, three *Mucor circinelloides*, one *M. indicus*, two isolates that could not be differentiated between *M. circinelloides* and *M. ramosissimus*, and two isolates that remained identified only as *Mucor* spp. Six of eight *Mucor* spp. isolates showed elevated isavuconazole MIC values (≥8 mg/L). However, one *Mucor* spp. and one *M. circinelloides*/*M*. *ramosissimus* exhibited isavuconazole MIC values of 2 and 4 mg/L, respectively. Amphotericin B was the most active antifungal agent against eight *Mucor* spp. isolates (MIC_50_, 0.5 mg/L; MIC range, 0.25–0.5 mg/L), followed by posaconazole (MIC_50_, 2 mg/L; MIC range, 0.5–>8 mg/L) and itraconazole (MIC_50_, 4 mg/L; MIC range, 2–8 mg/L). Voriconazole exhibited MIC_50_ value of >8 mg/L.

Four *Rhizomucor pusillus* and two *Syncephalastrum* spp. isolates, which corresponded to the six remaining isolates in the Mucorales order included in this study, exhibited variable isavuconazole MIC values. Isavuconazole was active against three *Rhizomucor pusillus* isolates (MIC, 2 mg/L) and one *Syncephalastrum* spp. (MIC, 2 mg/L) isolate. Isavuconazole (MIC_50_, 2 mg/L; MIC range, 2–>8 mg/L) showed similar activity to itraconazole (MIC_50_, 1 mg/L; MIC range, 0.5–4 mg/L) against four *Rhizomucor pusillus* isolates, and displayed four-fold higher MIC_50_ values than posaconazole (MIC_50_, 0.5 mg/L; MIC range, 0.5–1 mg/L) and amphotericin B (MIC_50_, 0.5 mg/L; MIC range, 0.25–0.5 mg/L). Only two isolates of *Syncephalastrum* spp. were recovered. Both isolates displayed lower MIC values for amphotericin B (MICs, 0.25–0.5 mg/L) and posaconazole (MICs, 0.5 mg/L) than itraconazole (MICs, 1–2 mg/L), isavuconazole (MICs, 2–>8 mg/L), and voriconazole (MICs, >8 mg/L).

## 4. Discussion

The first WHO fungal priority pathogens list was recently published. The Mucorales order is included in this list as a high priority due to the rising threat of infections, combined with existing and emerging resistance and treatability issues, and to promote research, development, and public health interventions [19]. Mucormycosis is one of the most common non-*Aspergillus* mould infections, plus it is a difficult-to-diagnose disease with high morbidity and mortality. All-cause mortality rates for mucormycosis range from 40% to 80%, depending on underlying conditions and the site of infection [20]. The most prevalent conditions associated with mucormycosis are diabetes mellitus, an immunocompromised status, especially when patients are receiving treatment for haematological malignancies or undergoing transplantation, and, more recently, SARS-CoV-2 infection [21]. The respiratory tract is the most frequent portal of entry, followed by the direct inoculation of organisms into disrupted skin. The characteristic angioinvasive ability of Mucorales organisms can lead to disseminated and fatal infections [22]. In this study, most Mucorales isolates were recovered from pneumonia in hospitalized patients and skin and skin structure infections, while other infection sites included the sinus and intra-abdominal area.

The Mucorales order includes a wide variety of genera; 38 different species have been reported to cause mucormycosis [23]. Similar to previous studies, our results showed that *Rhizopus* spp., *Mucor* spp., and *Lichtheimia* spp. were the most common Mucorales organisms recovered from Europe, USA, and Asia-Pacific [22,24,25]. Other Mucorales organisms include *Rhizomucor*, *Syncephalastrum*, *Cunninghamella*, *Apophysomyces*, and *Saksenaea* spp., but these genera are rarely isolated. Only four *Rhizomucor* and two *Syncephalastrum* isolates were included in the SENTRY Program between 2017 and 2020. However, new diagnostic tools, such as the detection of circulating Mucorales DNA by qPCR in serum, may change this epidemiology [26,27]. A recent study using Mucorales qPCR in serum showed a distribution of 35% of probable/proven cases due to *Mucor*/*Rhizopus*, 25% *Rhizomucor*, 20% *Lichtheimia*, and 10% a mixed infection by two Mucorales genera [27]. Notably, *Rhizomucor* DNA was identified in the serum of nine patients with negative cultures, suggesting this genus might be more difficult to recover from tissue cultures.

Although MALDI-TOF MS is increasingly used to identify of filamentous fungi, including Mucorales, ITS sequencing is the method of choice to identify Mucorales species [28]. Here, MALDI-TOF MS was able to identify 71.2%/63.5% of the isolates, while ITS and/or 28S DNA sequencing identified the remaining 28.8%/17.3% isolates to the genus/species level, respectively. Previous studies demonstrated that species/genus identification rates for 111 Mucorales isolates were 49.5%/66.7% and 81.1%/100% using Bruker Library v1.0 alone and in combination with an in-house library, respectively [29]. Notably, the Bruker MALDI-TOF MS filamentous library has been improved tremendously since its first release (v1.0)—from a total of 65 reference spectra (MSP) covering 25 genera and 41 species to 577 MSPs in the version 3.0, including 180 species and 10 strains only identified at genus level. These results show that while MALDI-TOF MS has revolutionized the identification of human pathogens, the Mucorales library still needs to be expanded. These libraries continue to be improved. The recently released Bruker MALDI-TOF MS filamentous library v4.0 covers 247 species/species groups, and 27 strains at genus level. Additionally, although ITS sequencing was sufficient to resolve most of the morphospecies, this method presents limitations for the *Mucor circinelloides* species complex and the *Syncephalastrum* genus [23,30]. In the *Mucor circinelloides* species complex, protein-coding genes such as *tsr1* or *rpb1* have a much higher power than ITS, but reference sequences for these genes are usually lacking [31]. In the genus *Syncephalastrum*, some strains have two clearly differing types of ITS sequences [30]. Additional sequencing targets may be required, such as D1–D2 domains of 28S rDNA. The 28S target has been used to resolve species identification within the Mucorales order. However, because mucormycosis is relatively rare and identification to the genus/species is difficult to achieve and not imperative to guide treatment, there are few medical centers that can accurately identify these organisms and perform susceptibility testing.

Antifungal susceptibility methods to test Mucorales isolates are published by both CLSI and the European Committee for Antimicrobial Susceptibility Testing (EUCAST), but these methods differ on the media used and the inoculum size [17,32]. Neither CLSI nor EUCAST have published clinical breakpoints against Mucorales due to the lack of data that can correlate MIC values and clinical outcomes. This lack of data is a result of how difficult it is to perform clinical studies of such a rare disease. As such, there are only a few prospective studies on the treatment of mucormycosis [12,33,34,35]. In the VITAL study, a Phase III single-arm, open label trial, the efficacy of isavuconazole treatment in adults with mucormycosis was similar to that of amphotericin B in case-matched controls [12]. Based on those results, isavuconazole was licensed by the US FDA for the primary treatment of invasive mucormycosis in adults [13]. The EMA also approved isavuconazole for the treatment of mucormycosis when treatment with amphotericin B is not appropriate [36].

The Mucorales organisms isolated from the VITAL study were similar to those recovered in this surveillance study but, in the VITAL study, one third of isolates did not have species differentiation [12]. Therefore, surveillance studies applying gold-standard methodologies are critical to the understanding of the epidemiology and activity of antifungal agents against the Mucorales order.

In our study, isavuconazole was active (MIC values, <4 mg/L) against 71.2% of the Mucorales collection, particularly against *R. microsporus* group (100% inhibited at ≤4 mg/L) and *Lichtheimia* spp. (72.7% inhibited at ≤4 mg/L), but displayed limited activity against *M. circinelloides*, corroborating the findings of other authors [37,38,39,40]. Overall, in this collection and previous studies, most of the isolates showed lower MIC values for amphotericin B and posaconazole than for isavuconazole. The clinical significance of this observation is unknown, as clinical breakpoints are not available for these drugs.

Pharmacokinetic and pharmacodynamic parameters (PK/PD) come into play when trying to extrapolate in vitro susceptibility data into the actual bioavailability and efficacy of the drug at the infection site. Data from PK/PD studies showed that, in healthy male volunteers, isavuconazole mean C_max_ values at a steady state were 2.61 and 2.55 μg/mL in plasma after oral and intravenous administration, respectively [41]. Similarly, posaconazole mean C_max_ values after a single intravenous 300-mg dose and on day 14 of treatment with 300 mg once daily (loading dose 300 mg b.i.d.) were 1.6 μg/mL and 2.6 μg/mL in plasma, respectively [42]. C_max_ values of 2 μg/mL were reached with amphotericin B lipid complex at standard doses [42]. Based on the in vitro activity, PK/PD, and available clinical evidence, the first-line treatment with liposomal amphotericin B, isavuconazole, or posaconazole are supported by the European Confederation of Medical Mycology and the International Society for Human and Animal Mycology (ECMM/ISHAM), the Australasian Antifungal Guidelines Steering Committee, and other scientific associations [1,9,10,43]. Isavuconazole and posaconazole are also recommended as mucormycosis salvage treatment [1,9,10,43]. Due to the antifungal activity variability observed in ours and previous reports among different Mucorales genera and species [37,38,39,40,44], we encourage clinical laboratories to perform species identification and provide antifungal susceptibility testing. Antifungal susceptibility testing is generally recommended for epidemiological purposes and to monitor for potential resistance development, but can also better inform clinical decisions, improving patient outcomes.

There are some limitations in this survey that must be acknowledged. First, due to the rarity of these infections, there are a limited number of isolates per species, and caution must be taken on extrapolating it to clinical practice. Second, we do not identify those patients who received an antifungal agent, nor collect clinical outcome data. As such, clinical correlation between MIC values and clinical outcomes were unable to be established. Finally, the SENTRY Surveillance Program is a sentinel, not a population-based surveillance.

In summary, isavuconazole, posaconazole, and amphotericin B exhibited activity against most Mucorales. Isavuconazole was notably active against *Rhizopus* spp., *Lichtheimia* spp., and *Rhizomucor* spp., although variability within genera and species was observed. These results support the recommendation to use isavuconazole as an alternative first-line or salvage therapy to treat Mucorales infections. Furthermore, continuous monitoring of the epidemiology and antifungal activity of these compounds against Mucorales is warranted. And, based on this data, clinical laboratories should pursue genera and species identification whenever possible and perform antifungal susceptibility testing for the first-line antifungal agents. However, it should be emphasized that no clinical data are available to validate breakpoints for any antifungal drug against the Mucorales.

## Figures and Tables

**Table 1:** Activity of isavuconazole and comparator agents against Mucorales isolates causing invasive infections worldwide (2017–2020).

Organism/Organism Group (No. of Isolates)	No. and Cumulative % of Isolates Inhibited at MIC (mg/L) of:								MIC_50_	MIC_90_
	≤0.12	0.25	0.5	1	2	4	8	> ^a^		
*Mucorales* order										
Isavuconazole (52)			0 0.0	14 26.9	17 59.6	6 71.2	4 78.8	11 100.0	2	>8
Itraconazole (52)		0 0.0	3 5.8	20 44.2	15 73.1	4 80.8	7 94.2	3 100.0	2	8
Voriconazole (52)					0 0.0	4 7.7	15 36.5	33 100.0	>8	>8
Posaconazole (52)	0 0.0	1 1.9	28 55.8	10 75.0	5 84.6	2 88.5	2 92.3	4 100.0	0.5	8
Amphotericin B (52)	0 0.0	5 9.6	31 69.2	15 98.1	1 100.0				0.5	1
*Lichtheimia* spp.										
Isavuconazole (11)				0 0.0	5 45.5	3 72.7	2 90.9	1 100.0	4	8
Itraconazole (11)			0 0.0	7 63.6	4 100.0				1	2
Voriconazole (11)							0 0.0	11 100.0	>8	>8
Posaconazole (11)		0 0.0	8 72.7	3 100.0					0.5	1
Amphotericin B (11)		0 0.0	9 81.8	2 100.0					0.5	1
*Mucor* spp.										
Isavuconazole (8)				0 0.0	1 12.5	1 25.0	1 37.5	5 100.0	>8	-
Itraconazole (8)				0 0.0	2 25.0	2 50.0	4 100.0		4	-
Voriconazole (8)							0 0.0	8 100.0	>8	-
Posaconazole (8)		0 0.0	1 12.5	2 37.5	2 62.5	0 62.5	1 75.0	2 100.0	2	-
Amphotericin B (8)	0 0.0	3 37.5	5 100.0						0.5	-
*Rhizomucor* spp.										
Isavuconazole (4)				0 0.0	3 75.0	0 75.0	0 75.0	1 100.0	2	-
Itraconazole (4)		0 0.0	1 25.0	2 75.0	0 75.0	1 100.0			1	-
Voriconazole (4)						0 0.0	1 25.0	3 100.0	>8	-
Posaconazole (4)		0 0.0	3 75.0	1 100.0					0.5	-
Amphotericin B (4)	0 0.0	1 25.0	3 100.0						0.5	-
*Rhizopus* spp.										
Isavuconazole (27)			0 0.0	14 51.9	7 77.8	2 85.2	1 88.9	3 100.0	1	>8
Itraconazole (27)		0 0.0	2 7.4	10 44.4	8 74.1	1 77.8	3 88.9	3 100.0	2	>8
Voriconazole (27)					0 0.0	4 14.8	14 66.7	9 100.0	8	>8
Posaconazole (27)	0 0.0	1 3.7	14 55.6	4 70.4	3 81.5	2 88.9	1 92.6	2 100.0	0.5	8
Amphotericin B (27)		0 0.0	13 48.1	13 96.3	1 100.0				1	1
*Syncephalastrum* spp.										
Isavuconazole (2)				0 0.0	1 50.0	0 50.0	0 50.0	1 100.0	2	-
Itraconazole (2)			0 0.0	1 50.0	1 100.0				1	-
Voriconazole (2)							0 0.0	2 100.0	>8	-
Posaconazole (2)		0 0.0	2 100.0						0.5	-
Amphotericin B (2)	0 0.0	1 50.0	1 100.0						0.25	-

^a^ Greater than the highest concentration tested.

**Table 2:** Isavuconazole and comparators activity against Mucorales group split by genera and species.

Organism	Antifungal Agent	No. of Isolates	MIC (mg/L)		
			50%	90%	Range
Mucorales					
	Isavuconazole	52	2	>8	1–>8
	Itraconazole	52	2	8	0.5->8
	Voriconazole	52	>8	>8	4–>8
	Posaconazole	52	0.5	8	0.25–>8
	Amphotericin B	52	0.5	1	0.25–2
All *Rhizopus* spp.					
	Isavuconazole	27	1	>8	1–>8
	Itraconazole	27	2	>8	0.5–>8
	Voriconazole	27	8	>8	4–>8
	Posaconazole	27	0.5	8	0.25–>8
	Amphotericin B	27	1	1	0.5–2
*R. microsporus* group					
	Isavuconazole	16	1	2	1–4
	Itraconazole	16	2	>8	0.5–>8
	Voriconazole	16	8	>8	4–>8
	Posaconazole	16	0.5	>8	0.25–>8
	Amphotericin B	16	1	1	0.5–1
*R. arrhizus* species complex					
	Isavuconazole	10	2	>8	1–>8
	Itraconazole	10	1	8	0.5–8
	Voriconazole	10	8	>8	4–>8
	Posaconazole	10	0.5	4	0.5–4
	Amphotericin B	10	0.5	1	0.5–1
All *Lichtheimia* spp.					
	Isavuconazole	11	4	8	2–>8
	Itraconazole	11	1	2	1–2
	Voriconazole	11	>8	>8	>8
	Posaconazole	11	0.5	1	0.5–1
	Amphotericin B	11	0.5	1	0.5–1
*L. corymbifera*					
	Isavuconazole	7	2	-	2–>8
	Itraconazole	7	1	-	1–2
	Voriconazole	7	>8	-	>8
	Posaconazole	7	0.5	-	0.5–1
	Amphotericin B	7	0.5	-	0.5–1
All *Mucor* spp.					
	Isavuconazole	8	>8	-	2–>8
	Itraconazole	8	4	-	2–8
	Voriconazole	8	>8	-	>8
	Posaconazole	8	2	-	0.5–>8
	Amphotericin B	8	0.5	-	0.25–0.5
*M. circinelloides*					
	Isavuconazole	3	>8	-	8–>8
	Itraconazole	3	8	-	2–8
	Voriconazole	3	>8	-	>8
	Posaconazole	3	2	-	0.5–>8
	Amphotericin B	3	0.5	-	0.5
*M. circinelloides/M. ramosissimus*					
	Isavuconazole	2	4	-	4–>8
	Itraconazole	2	2	-	2–4
	Voriconazole	2	>8	-	>8
	Posaconazole	2	2	-	2–8
	Amphotericin B	2	0.25	-	0.25
*Rhizomucor pusillus*					
	Isavuconazole	4	2	-	2–>8
	Itraconazole	4	1	-	0.5–4
	Voriconazole	4	>8	-	8–>8
	Posaconazole	4	0.5	-	0.5–1
	Amphotericin B	4	0.5	-	0.25–0.5
*Syncephalastrum* spp.					
	Isavuconazole	2	2	-	2–>8
	Itraconazole	2	1	-	1–2
	Voriconazole	2	>8	-	>8
	Posaconazole	2	0.5	-	0.5
	Amphotericin B	2	0.25	-	0.25–0.5

“-“, MIC_90_ not calculated due to the number of isolates (<10 isolates).

## Data Availability

The data presented in this study are available on request from the corresponding author. The data are not publicly available as it is proprietary.

## Supplementary Materials

The following supporting information can be downloaded at: https://www.mdpi.com/article/10.3390/jof9020241/s1, Table S1: Distribution of isolates per Mucorales genera and regions.
