# Supplementary material for: Antifungal Activity of Isavuconazole and Comparator Agents against Contemporaneous Mucorales Isolates from USA, Europe, and Asia-Pacific
Source: J Fungi (Basel). 2023 Feb 11;9(2):241. doi: 10.3390/jof9020241 (PMC9960003; doi:10.3390/jof9020241)
Supplement: Supplementary file 1 [file jof-09-00241-s001.zip › jof-2128221-supplementary.docx]

Supplement Material

Table S1. Distribution of isolates per Mucorales genus and regions

| Organisms | no. of isolates (% of isolates per genus) | | | Total no. of isolates per genus |
| --- | --- | --- | --- | --- |
|  | Europe | USA | Asia-Pacific |  |
| *Lichtheimia* spp. | 7 (30.4) | 3 (14.3) | 1 (12.5) | 11 |
| *Mucor* spp. | 3 (13.0) | 4 (19.0) | 1 (12.5) | 8 |
| *Rhizomucor* spp. | 1 (4.3) | 3 (14.3) | 0 (0.0) | 4 |
| *Rhizopus* spp. | 12 (52.2) | 10 (47.6) | 5 (62.5) | 27 |
| *Syncephalastrum* spp. | 0 (0.0) | 1 (4.8) | 1 (12.5) | 2 |
| Total | 23 | 21 | 8 | 52 |

Table S2 Activity of isavuconazole and comparator agents against Mucorales isolates causing invasive infections worldwide (2017-2020)

| **Organism/organism group (no. of isolates)** | **No. and cumulative % of isolates inhibited at MIC (mg/L) of:** | | | | | | | |  | **MIC_50_** | **MIC_90_** |
| --- | --- | --- | --- | --- | --- | --- | --- | --- | --- | --- | --- |
|  | **≤0.12** | **0.25** | **0.5** | **1** | **2** | **4** | **8** | **> ^a^** |  |  |  |
| *Mucorales* group | | | | | | | | | | |  |
| Isavuconazole (52) |  |  | 0 0.0 | 14 26.9 | 17 59.6 | 6 71.2 | 4 78.8 | 11 100.0 |  | 2 | >8 |
| Itraconazole (52) |  | 0 0.0 | 3 5.8 | 20 44.2 | 15 73.1 | 4 80.8 | 7 94.2 | 3 100.0 |  | 2 | 8 |
| Voriconazole (52) |  |  |  |  | 0 0.0 | 4 7.7 | 15 36.5 | 33 100.0 |  | >8 | >8 |
| Posaconazole (52) | 0 0.0 | 1 1.9 | 28 55.8 | 10 75.0 | 5 84.6 | 2 88.5 | 2 92.3 | 4 100.0 |  | 0.5 | 8 |
| Amphotericin B (52) | 0 0.0 | 5 9.6 | 31 69.2 | 15 98.1 | 1 100.0 |  |  |  |  | 0.5 | 1 |
|  | | | | | | | | | | |  |
| *Lichtheimia* spp. | | | | | | | | | | |  |
| Isavuconazole (11) |  |  |  | 0 0.0 | 5 45.5 | 3 72.7 | 2 90.9 | 1 100.0 |  | 4 | 8 |
| Itraconazole (11) |  |  | 0 0.0 | 7 63.6 | 4 100.0 |  |  |  |  | 1 | 2 |
| Voriconazole (11) |  |  |  |  |  |  | 0 0.0 | 11 100.0 |  | >8 | >8 |
| Posaconazole (11) |  | 0 0.0 | 8 72.7 | 3 100.0 |  |  |  |  |  | 0.5 | 1 |
| Amphotericin B (11) |  | 0 0.0 | 9 81.8 | 2 100.0 |  |  |  |  |  | 0.5 | 1 |
|  | | | | | | | | | | |  |
| *Mucor* spp. | | | | | | | | | | |  |
| Isavuconazole (8) |  |  |  | 0 0.0 | 1 12.5 | 1 25.0 | 1 37.5 | 5 100.0 |  | >8 | - |
| Itraconazole (8) |  |  |  | 0 0.0 | 2 25.0 | 2 50.0 | 4 100.0 |  |  | 4 | - |
| Voriconazole (8) |  |  |  |  |  |  | 0 0.0 | 8 100.0 |  | >8 | - |
| Posaconazole (8) |  | 0 0.0 | 1 12.5 | 2 37.5 | 2 62.5 | 0 62.5 | 1 75.0 | 2 100.0 |  | 2 | - |
| Amphotericin B (8) | 0 0.0 | 3 37.5 | 5 100.0 |  |  |  |  |  |  | 0.5 | - |
|  | | | | | | | | | | |  |
| *Rhizomucor* spp. | | | | | | | | | | |  |
| Isavuconazole (4) |  |  |  | 0 0.0 | 3 75.0 | 0 75.0 | 0 75.0 | 1 100.0 |  | 2 | - |
| Itraconazole (4) |  | 0 0.0 | 1 25.0 | 2 75.0 | 0 75.0 | 1 100.0 |  |  |  | 1 | - |
| Voriconazole (4) |  |  |  |  |  | 0 0.0 | 1 25.0 | 3 100.0 |  | >8 | - |
| Posaconazole (4) |  | 0 0.0 | 3 75.0 | 1 100.0 |  |  |  |  |  | 0.5 | - |
| Amphotericin B (4) | 0 0.0 | 1 25.0 | 3 100.0 |  |  |  |  |  |  | 0.5 | - |
|  | | | | | | | | | | |  |
| *Rhizopus* spp. | | | | | | | | | | |  |
| Isavuconazole (27) |  |  | 0 0.0 | 14 51.9 | 7 77.8 | 2 85.2 | 1 88.9 | 3 100.0 |  | 1 | >8 |
| Itraconazole (27) |  | 0 0.0 | 2 7.4 | 10 44.4 | 8 74.1 | 1 77.8 | 3 88.9 | 3 100.0 |  | 2 | >8 |
| Voriconazole (27) |  |  |  |  | 0 0.0 | 4 14.8 | 14 66.7 | 9 100.0 |  | 8 | >8 |
| Posaconazole (27) | 0 0.0 | 1 3.7 | 14 55.6 | 4 70.4 | 3 81.5 | 2 88.9 | 1 92.6 | 2 100.0 |  | 0.5 | 8 |
| Amphotericin B (27) |  | 0 0.0 | 13 48.1 | 13 96.3 | 1 100.0 |  |  |  |  | 1 | 1 |
|  | | | | | | | | | | |  |
| *Syncephalastrum* spp. | | | | | | | | | | |  |
| Isavuconazole (2) |  |  |  | 0 0.0 | 1 50.0 | 0 50.0 | 0 50.0 | 1 100.0 |  | 2 | - |
| Itraconazole (2) |  |  | 0 0.0 | 1 50.0 | 1 100.0 |  |  |  |  | 1 | - |
| Voriconazole (2) |  |  |  |  |  |  | 0 0.0 | 2 100.0 |  | >8 | - |
| Posaconazole (2) |  | 0 0.0 | 2 100.0 |  |  |  |  |  |  | 0.5 | - |
| Amphotericin B (2) | 0 0.0 | 1 50.0 | 1 100.0 |  |  |  |  |  |  | 0.25 | - |

^a^ Greater than the highest concentration tested.
